# Retraction: Combined *GSTM1-Null*, *GSTT1-Active*, *GSTA1 Low-Activity* and *GSTP1-Variant* Genotype Is Associated with Increased Risk of Clear Cell Renal Cell Carcinoma

**DOI:** 10.1371/journal.pone.0344546

**Published:** 2026-03-10

**Authors:** The PLOS One Editors

After this article [1] was published, the Ministry of Health of Republic of Serbia and the Ethics Board of Serbia contacted PLOS about concerns with the ethics approvals in [1] and requested the retraction of [1]. Specifically, a representative of the Ethics Board of Serbia stated that the study in [1] was conducted without the necessary approval from the Ethics Board of the health institution (the University Clinical Center of Serbia) whose patients and biological material are the subject of the research in [1].

The corresponding author stated that [1] was conducted with all the necessary approvals required at the time of sample collection and later data analysis, and provided copies of the ethics approval from the Ethics Committee of the Faculty of Medicine, University of Belgrade and a letter of permission from the Director of the Clinic of Urology, University Clinical Center of Serbia.

A representative of the University of Belgrade stated that [1] was conducted with all necessary ethical approvals from the Ethics Committee of the Faculty of Medicine, University of Belgrade which covered the collection of biological samples and data from patients at the Urology Clinic of the University Clinical Center of Serbia for the stated research purposes. They also stated that approval from the Ethics Committee of the Faculty of Medicine, University of Belgrade and a letter of permission from the Director of the Clinic of Urology, University Clinical Center of Serbia was compliant with the University of Belgrade's ethical standards at the time of sample collection and data analysis in [1]. The representative of the University of Belgrade stated that, at the time the study in [1] was conducted, discontinuities existed between the Law on Patients’ Rights and the University of Belgrade’s bylaws, and that the jurisdictions of the University of Belgrade and healthcare institutions, including the University Clinical Center of Serbia, were not clearly delineated.

A representative of the University Clinical Center of Serbia stated that the study in [1] was not reviewed and approved by the Ethics Board of the University Clinical Center of Serbia. They stated that the procedure for review and approval by the Ethics Committee of the Faculty of Medicine, University of Belgrade and written consent from the Director of the Clinic of Urology, University Clinical Center of Serbia was in accordance with the Rulebook on Doctoral Studies of the University of Belgrade, which did not include specifics in the case of research involving patients.

In light of concerns that the relevant approvals for clinical research involving patients were not in place at the time the study reported in [1] was conducted, and based on input from the Ethics Board of Serbia and the University Clinical Center of Serbia, the *PLOS One* Editors retract this article.

TDP agreed with the retraction. VMC, TPS, GMBJ, ARSR, MGM, DPD, TMR, and MSPE did not agree with the retraction. SMRS, LMB and ZMD either did not respond directly or could not be reached.

## References

[1] Coric VM, Simic TP, Pekmezovic TD, Basta-Jovanovic GM, Savic Radojevic AR, Radojevic-Skodric SM, et al. RETRACTED: Combined *GSTM1-Null, GSTT1-Active, GSTA1 Low-Activity* and *GSTP1-Variant* Genotype Is Associated with Increased Risk of Clear Cell Renal Cell Carcinoma. PLoS One. 2016;11(8):e0160570. doi: 10.1371/journal.pone.0160570 27500405 PMC4976979

